# A multi-regression framework to improve diagnostic ability of optical coherence tomography retinal biomarkers to discriminate mild cognitive impairment and Alzheimer’s disease

**DOI:** 10.1186/s13195-022-00982-0

**Published:** 2022-03-10

**Authors:** Jacqueline Chua, Chi Li, Lucius Kang Hua Ho, Damon Wong, Bingyao Tan, Xinwen Yao, Alfred Gan, Florian Schwarzhans, Gerhard Garhöfer, Chelvin C. A. Sng, Saima Hilal, Narayanaswamy Venketasubramanian, Carol Y. Cheung, Georg Fischer, Clemens Vass, Tien Yin Wong, Christopher Li-Hsian Chen, Leopold Schmetterer

**Affiliations:** 1 Singapore Eye Research Institute, Singapore National Eye Centre, 20 College Road, The Academia, Level 6, Discovery Tower, Singapore, 169856 Singapore; 2 Ophthalmology and Visual Sciences Academic Clinical Program, Duke-NUS Medical School, National University of Singapore, Singapore, Singapore; 3 SERI-NTU Advanced Ocular Engineering (STANCE), Singapore, Singapore; 4 School of Chemical and Biological Engineering, Nanyang Technological University, Singapore, Singapore; 5 Center for Medical Statistics Informatics and Intelligent Systems, Section for Medical Information Management, Medical University Vienna, Vienna, Austria; 6 Department of Clinical Pharmacology, Medical University Vienna, Vienna, Austria; 7 Department of Ophthalmology, National University Hospital, Singapore, Singapore; 8 Memory Aging and Cognition Centre, Departments of Pharmacology and Psychological Medicine, Yong Loo Lin School of Medicine, National University of Singapore, Singapore, Singapore; 9 Saw Swee Hock School of Public Health, National University of Singapore, Singapore, Singapore; 10 Raffles Neuroscience Centre, Raffles Hospital, Singapore, Singapore; 11 Department of Ophthalmology and Visual Sciences, The Chinese University of Hong Kong, Sha Tin, Hong Kong; 12 Department of Ophthalmology and Optometry, Medical University Vienna, Vienna, Austria; 13 Center for Medical Physics and Biomedical Engineering, Medical University Vienna, Vienna, Austria; 14 Institute of Molecular and Clinical Ophthalmology, Basel, Switzerland

**Keywords:** Optical coherence tomography, mild cognitive impairment, Alzheimer’s disease

## Abstract

**Background:**

Diagnostic performance of optical coherence tomography (OCT) to detect Alzheimer’s disease (AD) and mild cognitive impairment (MCI) remains limited. We assessed whether compensating the circumpapillary retinal nerve fiber layer (cpRNFL) thickness for multiple demographic and anatomical factors as well as the combination of macular layers improves the detection of MCI and AD.

**Methods:**

This cross-sectional study of 62 AD (*n* = 92 eyes), 108 MCI (*n* = 158 eyes), and 55 cognitively normal control (*n* = 86 eyes) participants. Macular ganglion cell complex (mGCC) thickness was extracted. Circumpapillary retinal nerve fiber layer (cpRNFL) measurement was compensated for several ocular factors. Thickness measurements and their corresponding areas under the receiver operating characteristic curves (AUCs) were compared between the groups. The main outcome measure was OCT thickness measurements.

**Results:**

Participants with MCI/AD showed significantly thinner measured and compensated cpRNFL, mGCC, and altered retinal vessel density (*p* < 0.05). Compensated RNFL outperformed measured RNFL for discrimination of MCI/AD (AUC = 0.74 vs 0.69; *p* = 0.026). Combining macular and compensated cpRNFL parameters provided the best detection of MCI/AD (AUC = 0.80 vs 0.69; *p* < 0.001).

**Conclusions and relevance:**

Accounting for interindividual variations of ocular anatomical features in cpRNFL measurements and incorporating macular information may improve the identification of high-risk individuals with early cognitive impairment.

**Supplementary Information:**

The online version contains supplementary material available at 10.1186/s13195-022-00982-0.

## Introduction

Alzheimer’s disease (AD) and other causes of dementia are set to rise worldwide and negatively affect patients as well as their families [1]. Given the similarities between the cortex and the retina, as well as a body of work supporting the connection between retinal and cerebral changes in AD [2, 3], there has been active investigation into use of the optical coherence tomography (OCT) to distinguish between symptomatic AD and/or mild cognitive impairment (MCI) and cognitively normal older adults [4].

Several studies have explored the relationship between OCT parameters in patients with MCI and AD. A recent meta-analysis reported that patients with AD have a thinner circumpapillary retinal nerve fiber layer (cpRNFL) and ganglion cell–inner plexiform layer (GC-IPL) compared to controls [2]. However, inconsistencies exist for cpRNFL thickness, where some studies have shown a reduction in cpRNFL thickness in AD patients [5–13], while others have not found a significant difference between AD patients and controls [14–19]. The high inter-individual variability of the cpRNFL thickness may partly explain the discordant results. The cpRNFL thickness measurement is influenced by several individual specific factors, such as age [20, 21], ethnicity [22, 23], and ocular anatomical features (e.g., retinal vessel profile [24–26]).

We recently developed a regression-based model (multi-regression) from normal individuals to compensate cpRNFL thickness for numerous factors [21, 27]. Our newly compensated cpRNFL thickness demonstrated a smaller standard deviation (SD) in comparison to conventional analysis of cpRNFL [21, 27]. This increases precision when comparing data to the normative database, while accounting for individual differences. We now extend this work to determine the discriminative ability of cpRNFL thickness to detect MCI and AD after compensating for ocular anatomical features. We hypothesize that the compensated RNFL thickness can lower the variability, leading to improvement in sensitivity and specificity.

While OCT parameters have been studied individually [2], none has attempted to combine these parameters to differentiate persons with cognitive impairment from those with no cognitive impairment. The secondary aim of the present study was to assess whether combining cpRNFL thickness and macular layers could further improve the differentiation of those with MCI and AD from healthy controls.

## Methods

### Study participants

This is a cross-sectional study, comprising participants aged 50 years and above, enrolled from a memory clinic, from January 2010 to February 2020 [28]. Dementia was diagnosed clinically following the Diagnostic and Statistical Manual of Mental Disorders (DSM)-IV criteria. The etiological diagnosis of AD was made using the National Institute of Neurological and Communicative Disorders and Stroke and the Alzheimer’s Disease and Related Disorders Association (NINCDS-ADRDA) [29]. MCI participants were defined as following the Peterson’s criteria and did not have impairment in activities of daily living. Cognitively normal controls attended the same clinic but were not impaired in any of the tested domains. Participants were excluded from this study if they were hypoxic, anoxic, hypotensive, hypertensive, uremic or hepatic encephalopathy, had traumatic, nutritional or toxic disorder affecting the central nervous system (CNS), any current or past substance abuse disorder that has affected the CNS, had intracerebral hemorrhage, cranial arteritis, CNS inflammatory vasculitis, moyamoya disease, CNS infection, space occupying intracranial mass lesion, obstructive or normal pressure hydrocephalus, difficulty in controlling epilepsy, medical illness requiring concomitant corticosteroid or immunosuppressant therapy, moribund state, and significant aphasia or dysarthria that will significantly impede cognitive assessment [30]. We excluded participants with glaucoma, vascular or nonvascular retinopathies, and age-related macular degeneration (AMD) from fundus photographs. AMD was defined according to the Age-Related Eye Disease Study grading system as the presence of drusen or/and pigment changes within the macula center [31].

All participants underwent detailed clinical assessment including administration of the clinical dementia rating (CDR) scale and neuropsychological assessments [29]. Trained research psychologists administered brief cognitive tests: the Mini-Mental State Examination (MMSE) and the Montreal Cognitive Assessment (MoCA) and a formal detailed neuropsychological test battery that has been locally validated in Singapore. This battery assesses seven domains, five of which are non-memory domains. The non-memory domains tested were as follows: Executive function: Frontal Assessment Battery and Maze Task; Attention: Digit Span, Visual Memory Span, and Auditory Detection; Language: 15-item Boston Naming Test and Verbal Fluency; Visuomotor speed: Symbol Digit Modality Test, Digit Cancellation; and Visuoconstruction: Wechsler Memory Scale-Revised (WMS-R) Visual Reproduction Copy task, Clock Drawing, and Weschler Adult Intelligence Scale-Revised (WAIS-R) subtest of Block Design. The memory domains tested were as follows: Verbal memory: Word List Recall and Story Recall; and Visual memory: Picture Recall and WMS-R Visual Reproduction [32].

Medical histories (e.g., for diabetes and hypertension) were collected, and seated blood pressure (BP) measurements were taken using an automated device during their clinical visits. This study was approved by the National Healthcare Group Domain Specific Review Board and the conduct of the study adhered to the Declaration of Helsinki. All participants or their primary caregivers gave written informed consent.

### Ocular examinations

All participants underwent standardized eye examinations, including auto-refraction-keratometry (Canon RK-5 Autorefractor Keratometer; Canon Inc., Tokyo, Japan) [33], intraocular pressure measurement (IOP), retinal photography with a nonmydriatic digital camera, and OCT imaging (see later section). Spherical equivalent (SE) was calculated as the spherical value plus half of the negative cylinder value. Two retinal fundus photographs with one centered at the optic disc and another centered at the macula were obtained to document the absence of eye diseases.

### Optical coherence tomography imaging

**Fig. 1:**
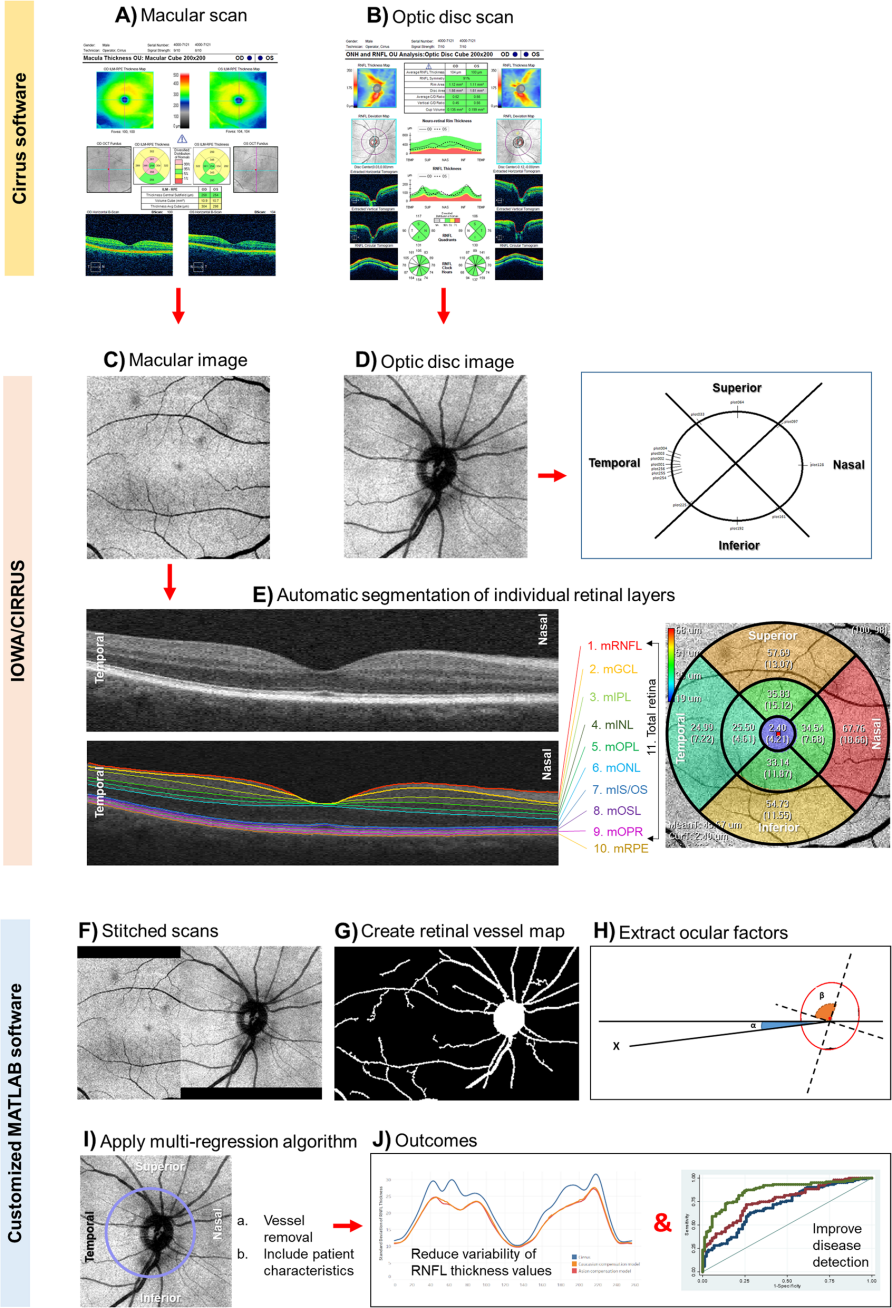
Steps to account for ocular factors from circumpapillary retinal nerve fiber layer (cpRNFL) measurement. **A**, **B** Capture the optical coherence tomography (OCT) scan protocols using Cirrus (Zeiss) system, one centered in the macula and the other centered in the optic disc. **C**–**E** Extract the cpRNFL measurements using Cirrus Review software and the individual macular layers using Iowa Reference Algorithms version 3.8.0 of the OCT layer segmentation program. **F** Register and stitch the macular and optic disc images. **G** Segment the retinal vessels to obtain the vessel tree. **H** Extract the optic disc and fovea features. **I** Calculate the cpRNFL retinal thickness, using a multi-regression compensation model. **J** Finally, the ideal model would reduce the variability of cpRNFL thickness measurements and/or improve disease detection

OCT scans were performed using the Cirrus spectral domain-OCT (Carl Zeiss Meditec, Inc., Dublin, CA, USA). Two different scan protocols were acquired, one centered on the macula and the other centered on the optic disc (200 A-scans × 200 B-scans; 6 × 6 mm; Fig. 1A–D) [21]. One trained grader, masked to the participant’s characteristics, reviewed the quality of all OCT datasets. Eyes with poor quality images (signal strength less than 6 and/or excessive movement artifacts and/or inconsistent signal intensity across the scan and/or segmentation failure) and missing variables were excluded from the analysis. Both eyes of each participant were included in this study according to the eligibility criteria described.

### Automated analysis of retinal thickness

The circumpapillary retinal nerve fiber layer (cpRNFL) thickness measurements were extracted from the Cirrus Review Software (software version 11.0.0.29946; Fig. 1D). The cpRNFL thickness measurement was obtained over four quadrants (superior, inferior, nasal, and temporal) that formed a 3.4-mm ring around the optic disc center, as well as the overall global thickness. Mean retinal thickness values of 10 retinal layers of the macular scan were extracted using the automatic OCT layer segmentation algorithm (Retinal Image Analysis Lab, Iowa Institute for Biomedical Imaging, Iowa City, IA; Fig. 1C, E) [34–36]. This software automatically segmented the OCT volume by delimiting the macular retinal nerve fiber layer (mRNFL), macular ganglion cell layer (mGCL), and macular inner plexiform layer (mIPL). We also computed the macular ganglion cell complex (mGCC; combining mRNFL, mGCL, and mIPL) and the mGCL plus mIPL (mGC-IPL). We extracted the retinal layer thickness measurements from the 9 areas as defined by the Early Treatment Diabetic Retinopathy Study (ETDRS) protocol [37]. All B-scans were checked for alignment and segmentation errors to confirm the accuracy of retinal thickness measurements [38].

### Automated extraction of ocular parameters

We automatically extracted several parameters using MATLAB (MathWorks Inc., R2018b, Natick, MA) and segmented the retinal vessel map from the OCT volumetric data (Fig. 1F, G) [27]. We integrated all the vessels within a band of diameter around the center of the optic disc, extending from 3.28–3.64 mm into a 256-sector retinal vessel density [39]. Optic disc parameters including its area, orientation (angle between the horizontal axis and the major axis of the optic disc), and ratio (quotient between major and minor axis) were extracted from the spectral-domain-OCT. From the stitched optic disc and macular image and considering fovea center as automatically determined in SD-OCT, we obtained two fovea parameters: first, the fovea distance, which corresponds to the distance between optic disc and fovea centers; second, the fovea angle, which corresponds to the angle between a line connecting fovea and optic disc centers and a horizontal line passing through the optic disc center (Fig. 1H).

### Compensation model

We previously generated compensated cpRNFL thickness based on the optic disc (ratio, orientation, and area), fovea (distance and angle), retinal vessel density, refractive error, and age (Fig. 1I, J) [21]. Retinal vessel density correlated weakly with measured cpRNFL thickness (*r* = 0.21, *p* < 0.001), which highlights the contribution that retinal blood vessels have on measured RNFL thickness (Additional file 1: Fig. S1). The relationship between retinal vessel density and compensated cpRNFL thickness was non-significant, which shows the effectivity of our compensation model (*r* = -0.08, *p* = 0.139; Additional file 1: Fig. S1). For the current study, we added 2 new parameters: ethnicity and signal strength since these are important determinants of cpRNFL thickness measurements [37, 40]. Model selection was performed by minimizing the Akaike Information Criteria (AIC), which gives a tradeoff between data fitting and model complexity, by estimating the expected loss in information in choosing a model [41]. After obtaining the best model in each sector, we retrieved the regression coefficients of the multivariate linear regression of that model.

### Statistical analyses

**Fig. 2:**
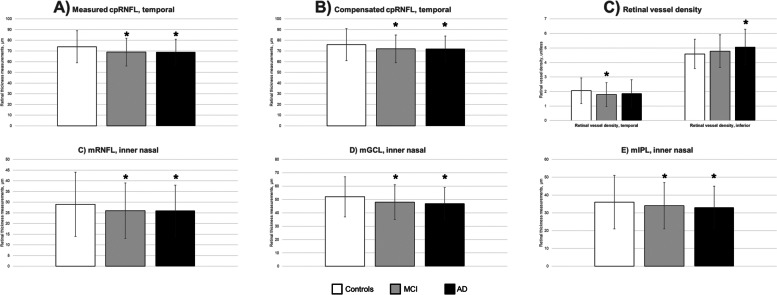
Comparison of thickness measurements of **A** measured circumpapillary retinal nerve fiber layer (cpRNFL), **B** compensated cpRNFL, **C** retinal vessel density, **D**, **F** macular layers in retinal nerve fiber layer (mRNFL), ganglion cell layer (mGCL), and inner plexiform layer (mIPL) in cognitively normal controls, mild cognitive impairment (MCI) cases, and Alzheimer’s disease (AD) cases. The data are adjusted for diabetes status, refractive error and represent mean ± standard deviation thickness in micrometers. Statistically significant results are indicated by an asterisk when compared to controls

We did a post hoc power calculation to evaluate the statistical power of current existing study (*n* = 170 MCI/AD cases vs 55 controls) using the means and standard deviations derived from the current study. For cpRNFL thickness (69 ± 12 μm vs 74 ± 15 μm), using an alpha error of 5%, we would have a post-hoc power of 66.4%. For the mGCIPL, using 33 ± 4 μm vs 36 ± 5 μm, derived from Fig. 2, we would have a post-hoc power of 98.2% (https://clincalc.com/stats/Power.aspx) [42].

To compare the characteristics of participants among groups, a one-way analysis of variance (ANOVA) was performed for continuous variables and chi-square tests were performed for categorical variables. Variables were included in a multivariable binary logistic regression model comparing MCI and AD versus healthy controls, MCI versus healthy controls, and lastly AD versus healthy controls. The diagnostic accuracy of the measured cpRNFL thickness (reference group), compensated cpRNFL thickness, and individual macular retinal layer measurements in differentiating cognitively normal controls between MCI and AD, or MCI, or AD were compared using the area under the receiver operating characteristics (ROC) curve (AUC). Clustered bootstrapping was used for inference to account for correlation among observations (eyes) for the same individual. The curves show the values at different levels of sensitivity (true positive rate) on the *y*-axis and 1-specificity (false-positive rate) on the *x*-axis for each parameter. The AUC summarizes the global value of the parameter, where values closer to 1 represent higher diagnostic discriminant ability. The sensitivity at 80% specificity was also calculated. The ROC curves and corresponding AUCs for retinal thickness were compared using the DeLong test for the difference between AUCs [43]. *P* value < 0.05 was considered statistically significant. All analyses were 2-tailed. Data were analyzed with statistical software (STATA, version 16; StataCorp LLC, College Station, USA).

## Results

Additional file 2: Fig. S2 detailed the inclusion and exclusion criteria of the study participants. Of the 466 enrolled participants, we excluded participants with poor quality scans (*n* = 91), presence of eye diseases (*n* = 125), and poor retinal segmentations (*n* = 24), leaving 55 healthy controls (*n* = 86 eyes), 108 MCI (*n* = 158 eyes), and 62 AD (*n* = 92 eyes) participants with good quality OCT who were free from eye diseases for analysis.

There was no significant difference in age and gender among the groups (Table 1). The mean age of participants was 72.8 ± 6.8 years and 57% were females. Compared with cognitively normal controls, persons with MCI/AD were more likely to have higher CDR scores (*p* < 0.001), higher prevalence of diabetes (*p* = 0.031), and more hyperopic refractive error (*p* < 0.001). There was no significant difference in the hypertension status (*p* = 0.111), systolic (*p* = 0.113), and diastolic (*p* = 0.304) blood pressure levels between groups. Ocular factors such as optic disc (*p* = 0.053) and macular (*p* = 0.644) scan qualities, optic disc area (*p* = 0.083), optic disc ratio (*p* = 0.661), optic disc orientation (*p* = 0.741), fovea distance (*p* = 0.935), and fovea angle (*p* = 0.846) did not differ between groups.

**Table 1:** Demographics and ocular characteristics of mild cognitive impairment (MCI) cases, Alzheimer’s disease (AD) cases, and cognitively normal controls

Characteristics	Controls (***n*** = 55)	MCI (***n*** = 108)	AD (***n*** = 62)	***p*** value
Age	71.0 ± 4.7	73.4 ± 6.3	73.3 ± 8.7	0.078
Gender, female	31 (56)	58 (54)	39 (63)	0.505
Diabetes, yes	8 (15)	35 (32)	21 (34)	**0.031**
Hypertension, yes	31 (56)	61 (57)	44 (72)	0.111
Systolic blood pressure, mmHg	137 ± 16	142 ± 16	142 ± 19	0.113
Diastolic blood pressure, mmHg	73 ± 9	74 ± 10	71 ± 11	0.304
Global CDR Score	0.11 ± 0.21	0.32 ± 0.24	1.17 ± 0.41	**< 0.001**
Signal strength, optic disc	7.56 ± 1.05	7.64 ± 1.13	7.92 ± 1.02	0.053
Signal strength, macular	7.81 ± 1.00	7.89 ± 1.15	7.97 ± 1.05	0.644
Optic disc area, mm^2^	1.86 ± 0.39	1.97 ± 0.39	1.98 ± 0.35	0.083
Optic disc ratio	1.13 ± 0.09	1.14 ± 0.08	1.13 ± 0.07	0.661
Optic disc orientation, degrees	95.85 ± 33.15	97.23 ± 31.15	99.51 ± 32.77	0.741
Fovea distance, μm	4.52 ± 0.29	4.53 ± 0.29	4.53 ± 0.28	0.935
Fovea angle, degrees	− 8.06 ± 3.95	− 7.74 ± 4.20	− 7.83 ± 4.34	0.846
Spherical equivalent refractive error, dioptres	− 1.11 ± 2.76	0.03 ± 1.88	− 0.06 ± 1.67	**< 0.001**

*AD* Alzheimer’s disease, *MCI* mild cognitive impairment, *CDR* Clinical Dementia Rating scale

Data presented are mean (SD) or number (%), as appropriate

*p value was obtained with ANOVA for the continuous variables and with chi-square tests for categorical variables

After adjusting for diabetes and refractive error, measured cpRNFL thickness in the temporal quadrant was significantly thinner in the MCI (69 ± 13 μm) and AD (69 ± 12 μm) groups than the normal controls (74 ± 15 μm; *p* = 0.048; Fig. 2A). Compensated cpRNFL thickness in the temporal quadrant was also significantly thinner in the MCI/AD participants than controls (*p* = 0.035; Fig. 2B). In the MCI group, retinal vessels around the optic disc were sparser in the temporal quadrant (1.79 ± 0.82 vs 2.05 ± 0.88; *p* = 0.030), and in the AD group, retinal vessels around the optic disc were denser in the inferior quadrant (5.07 ± 1.22 vs 4.59 ± 1.01; *p* = 0.031), as compared to controls (Fig. 2C).

For macular layers, mRNFL was significantly thinner in the MCI (inner and outer sectors except the temporal; *p* = 0.001) and AD (in all the sectors studied except the outer temporal; *p* = 0.002; Fig. 2D). mGCL was significantly thinner in the MCI (fovea and inner nasal; *p* = 0.010) and AD (fovea and all the inner sectors except the superior sector; *p* = 0.0106; Fig. 2E). mIPL was significantly thinner in the inner nasal region in both the MCI (34 ± 4 μm; *p* = 0.030) and AD (33 ± 4 μm; *p* = 0.012) groups compared to the normal controls (36 ± 5 μm; Fig. 2F). There were no statistical differences in the remaining layers between the group (Additional file 3: Fig. S3). As expected, total retina thickness was significantly thinner in both AD/MCI groups globally (Additional file 3: Fig. S3). Global total retinal thicknesses in AD (275 ± 15 μm; *p* = 0.027) and MCI (277 ± 17 μm; *p* = 0.042) groups were significantly lower than normal controls (282 ± 19 μm; *p* for trend = 0.030).

**Fig. 3:**
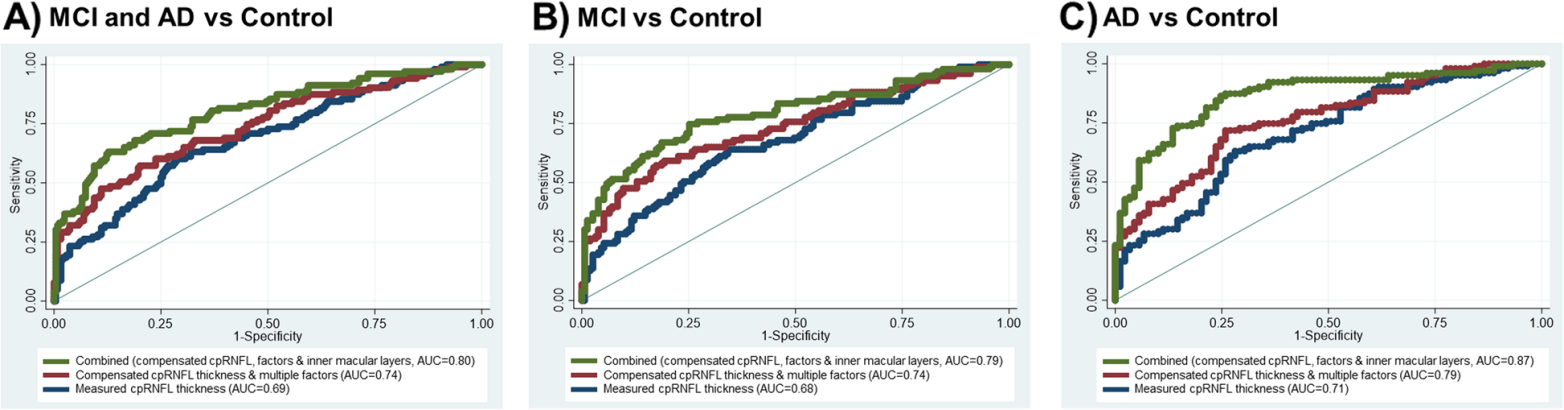
Receiver operating characteristic (ROC) curves and corresponding areas under the ROC curve (AUC) of measured cpRNFL thickness (measurement extracted directly from Cirrus), compensated cpRNFL thickness and multiple factors, and combined (compensated cpRNFL thickness, factors and inner macular layers) to discriminate **A** mild cognitive impairment (MCI) and Alzheimer’s disease (AD), **B** MCI, and **C** AD. Circumpapillary retinal nerve fiber layer (cpRNFL), inner macular layers represent RNFL (mRNFL), ganglion cell layer (mGCL), and inner plexiform layer (mIPL)

We next examined the diagnostic performance of the cpRNFL, retinal vessel density, and the macular layers (mRNFL, mGCL, and mIPL) to differentiate MCI/AD from controls (Table 2). There was no statistical significance between AUCs for measured cpRNFL thickness, retinal vessel density, and macular layers measurement for MCI/AD (*p* = 0.080). Compensated RNFL outperformed measured RNFL for discrimination of MCI (AUC = 0.74 vs 0.68; *p* = 0.020) and AD (AUC = 0.79 vs 0.71; *p* = 0.025; Fig. 3). mGCC outperformed mGC-IPL for discrimination of MCI (AUC = 0.71 vs 0.66; *p* = 0.038) whereas they were statistically insignificant for AD (AUC = 0.76 vs 0.75; *p* = 0.116). We selected mGCC as the macular parameter to be incorporated in the combined model with compensated cpRNFL, where it further improved the detection of MCI (AUC = 0.79 vs 0.68; *p* < 0.001) and AD (AUC = 0.87 vs 0.71; *p* < 0.001; Fig. 3).

**Table 2:** Diagnostic performance for discriminating mild cognitive impairment (MCI) and Alzheimer’s disease (AD), MCI, and AD from cognitively normal controls

No.	Parameter	Area under the receiver operating characteristic curve (95% confidence interval)	Sensitivity at 80% specificity	***p*** value
**A) MCI and AD vs control**				
1	Measured cpRNFL thickness	0.69 (0.62–0.75)	42.7	Ref
2	Retinal vessel density	0.61 (0.55–0.68)	35.9	0.080
3	Macular layers (mRNFL, mGCL and mIPL)	0.73 (0.68–0.79)	51.5	0.189
4	Compensated cpRNFL thickness and multiple ocular factors	0.74 (0.68–0.80)	56.3	**0.026**
5	Combined (#3 and #4)	0.80 (0.75–0.86)	68.0	**< 0.001**
**B) MCI vs control**				
1	Measured cpRNFL thickness	0.68 (0.61–0.74)	42.7	Ref
2	Retinal vessel density	0.60 (0.53–0.67)	34.0	0.092
3	Macular layers (mRNFL, mGCL and mIPL)	0.71 (0.65–0.78)	45.6	0.393
4	Compensated cpRNFL thickness and multiple ocular factors	0.74 (0.67–0.80)	59.2	**0.020**
5	Combined (#3 and #4)	0.79 (0.73–0.85)	67.0	**< 0.001**
**C) AD vs control**				
1	Measured cpRNFL thickness	0.71 (0.63–0.78)	36.9	Ref
2	Retinal vessel density	0.64 (0.56–0.72)	30.1	0.225
3	Macular layers (mRNFL, mGCL and mIPL)	0.76 (0.70–0.83)	56.3	0.086
4	Compensated cpRNFL thickness and multiple ocular factors	0.79 (0.72–0.85)	56.3	**0.025**
5	Combined (#3 and #4)	0.87 (0.82–0.92)	74.8	**< 0.001**
**D) MCI vs AD**				
1	Measured cpRNFL thickness	0.58 (0.51–0.66)	26.9	Ref
2	Retinal vessel density	0.59 (0.51–0.67)	36.3	0.916
3	Macular layers (mRNFL, mGCL and mIPL)	0.66 (0.58–0.74)	37.2	0.129
4	Compensated cpRNFL thickness and multiple ocular factors	0.64 (0.56–0.71)	35.9	0.112
5	Combined (#3 and #4)	0.72 (0.65–0.79)	42.1	**0.003**

Results for sensitivity is expressed as percentages. *P* value indicates the paired comparisons with the best parameter (reference group)

*cpRNFL* circumpapillary retinal nerve fiber layer, *mRNFL* macular retinal nerve fiber layer, *mGCL* macular ganglion cell layer, *mIPL* macular inner plexiform layer

We further calculated the model performance for distinguishing MCI from AD. There was no statistical significance between AUCs for measured cpRNFL thickness, retinal vessel density, macular layers measurement, and compensated RNFL in distinguishing MCI from AD (*p* = 0.112). Combining mGCC with either compensated (AUC = 0.72; *p* = 0.003; Table 2) or measured RNFL (AUC = 0.68; *p* = 0.023) significantly improved the detection of MCI from AD as compared to using measured cpRNFL thickness (AUC = 0.58) alone.

## Discussion

This case control study found that compensated cpRNFL thickness measurements were superior to conventional cpRNFL thickness analysis for distinguishing between normal and AD/MCI participants. Of note, the combination of mGCC and compensated cpRNFL showed the highest diagnostic capability to distinguish controls from MCI/AD. Our study adds further to a rapidly emerging field in using OCT as a retinal biomarker for AD by demonstrating that accounting for the cpRNFL thickness measurements for ocular anatomical variations as well as integrating information from inner retinal macular layers may allow for the improved identification of high-risk individuals with early cognitive impairment and dementia.

Current commercial OCT systems provide an objective quantification of the subject’s cpRNFL thickness measurements which is subsequently compared to their population-wide norm (also known as normative databases) [44]. For comparison, the inbuilt OCT software accounts the cpRNFL thickness measurements for age but not for ocular factors such as optic disc (size and area), disc-fovea angle, and retinal vessel position [24–26]. Our compensation model comprises precise alignment of the scans, adjust vessel profile, and input of patient’s anatomical features to OCT scans before analysis (Fig. 1). The subject’s compensated RNFL thickness would then be accounted for the influence of these ocular anatomical features, reducing measurement variability, and thus allowing the detection of smaller changes related to dementia when compared to the normative database. As demonstrated, our proposed compensation model resulted in an improvement in the diagnostic separation of controls from MCI/AD.

Is there a need to apply the compensation model to cpRNFL thickness measurements when using OCT in longitudinal monitoring of eye-brain functional changes? Retinal microcirculation is stated to decrease during normal aging, mainly in the narrowing of retinal arteriolar diameters [45]. Of note, we reported previously that the age-dependent thinning of cpRNFL thickness was largely related to the narrowing of retinal vessels rather than the loss of retinal ganglion cell axons [21]. Since the current OCT system is not able to differentiate retinal vessels from neuronal axons, the cpRNFL thickness measurement will include retinal vessels (Additional file 1: Fig. S1). We previously showed an altered retinal microvascular dysfunction in the eyes of patients with MCI/AD [28, 46]. Hence, the age-related narrowing of retinal vessels should be accounted for when using multiple cpRNFL thickness measures over time.

The topographic region-based analysis of the RNFL revealed thinning in the temporal zone of cpRNFL (in subjects with both MCI and AD). At the level of the macula, the nerve fiber layer is thinner in the nasal region, which is closer to the optic disc (Additional file 3: Fig. S3). For the remaining ganglion cell parameters, differences were found in the inner nasal region. Another study performed macular intra-retinal layer thickness-based analysis and found thinning as well as a thickening in almost all retinal layers in a small cohort of 18 AD individuals and 24 healthy controls [47]. However, they did not adjust for potential confounders such as diabetes. Considering our results, we speculate the first detectable changes of AD may occur at the level of the RNFL containing the ganglion cell axons, whereas modifications in the layer containing ganglion cell bodies (GCL) and dendrites (IPL) may take place at a later stage. This rationale is in support of compelling evidence that indicates that axonal degeneration occurs before cell body death in AD [48].

Additionally, the sector with greater damage (in subjects with both MCI and AD) is the temporal cpRNFL quadrant and the nasal mRNFL region, which coincide with the papillomacular bundle. Our results are consistent with authors who have found a thinner nasal macular region in patients with AD compared to controls [49, 50]. Melanopsin retinal ganglion cells are more concentrated in the parafoveal region, which generates the papillomacular bundle [51]. These melanopsin retinal ganglion cells are affected in postmortem eyes of AD cases [11].

Previous studies reported a higher diagnostic value of the macular retinal nerve fiber layer (mRNFL) than the circumpapillary RNFL (cpRNFL) region in AD neurodegeneration [52]. This is counterintuitive since the macular region reflects ~ 50% of the retinal nerve ganglion cells (RGCs) in the scanned area in comparison with the complete representation of the RGCs axons sampled by the cpRNFL scan [53]. One likely explanation is the high inter-individual variation of the cpRNFL thickness. After accounting for multiple factors, the compensated cpRNFL thickness demonstrated a higher diagnostic value in MCI/AD than combined macular layers (although not statistically significant).

### Strengths and limitations

Strengths of this study include a well-phenotyped cohort of AD and MCI individuals who were diagnosed according to internationally accepted criteria and a standardized study methodology. Also, we considered potential confounders of retinal thickness measurements such as age, gender, diabetes, blood pressure levels, signal strength, and refractive error. Our study has several limitations. First, our relatively small sample size was due to the exclusion of participants with ocular diseases, which expectedly increases with age. Second, cpRNFL thinning can be associated with pre-perimetric glaucoma. Nevertheless, we excluded all participants with glaucomatous optic disc pathology via fundus photography. Finally, this study was restricted to Asians; therefore, the generalizability of our results to persons of non-Asian ethnicities may be limited.

## Conclusions

In summary, our study shows that by combining macular ganglion cell complex with compensated cpRNFL thickness measurements for the variations of ocular anatomical features, the clinical utility of OCT to distinguish controls from MCI/AD is enhanced. The significant improvements in the diagnostic accuracy of MCI/AD resulting from these strategies are particularly important to improve the potential application of OCT on screening for cognitive impairment and dementia.

## Supplementary Information

**Additional file 1.**

**Additional file 2.**

**Additional file 3.**

## Data Availability

The datasets used and/or analyzed during the current study are available from the corresponding authors on reasonable request.
