# Supplementary material for: A multi-regression framework to improve diagnostic ability of optical coherence tomography retinal biomarkers to discriminate mild cognitive impairment and Alzheimer’s disease
Source: Alzheimers Res Ther. 2022 Mar 10;14:41. doi: 10.1186/s13195-022-00982-0 (PMC8908577; doi:10.1186/s13195-022-00982-0)
Supplement: Supplementary file 1 — Additional file 1. [file 13195_2022_982_MOESM1_ESM.pdf]

Additional file:

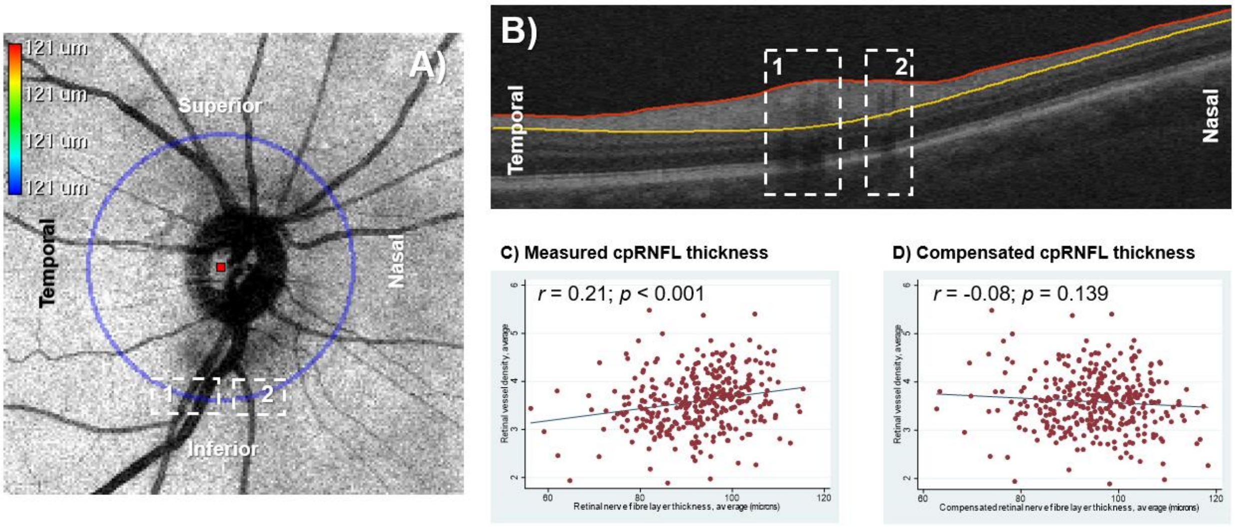

Additional file 1: **Figure S1:** An example of the right eye of a participant, showing the relation of circumpapillary retinal nerve fiber layer thickness (cpRNFL) and retinal vessels around the optic disc region in A) projection image. Retinal blood vessels are embedded within the RNFL thickness and therefore contribute to the RNFL thickness. The boundaries of retinal blood vessels may be detected by following their optical shadows cast onto the outer retina as seen from the B) OCT B-scan. The white dotted boxes show two retinal vessels within box #1 and one retinal vessel in box #2. C) Pearson's correlation was used to assess the relationship between cpRNFL and retinal vessel density. There was a small positive correlation between Cirrus cpRNFL thickness and retinal vessel density, $r=0.21$, $p<0.001$, with retinal vessel density explaining 4% of the variation in cpRNFL thickness. D) This relationship between cpRNFL thickness and retinal vessel density weakened and was no longer statistically significant after cpRNFL thickness was compensated to account for retinal vessel density ($r=-0.08$, $p=0.139$).
