# Supplementary material for: A multi-regression framework to improve diagnostic ability of optical coherence tomography retinal biomarkers to discriminate mild cognitive impairment and Alzheimer’s disease
Source: Alzheimers Res Ther. 2022 Mar 10;14:41. doi: 10.1186/s13195-022-00982-0 (PMC8908577; doi:10.1186/s13195-022-00982-0)
Supplement: Supplementary file 2 — Additional file 2. [file 13195_2022_982_MOESM2_ESM.pdf]

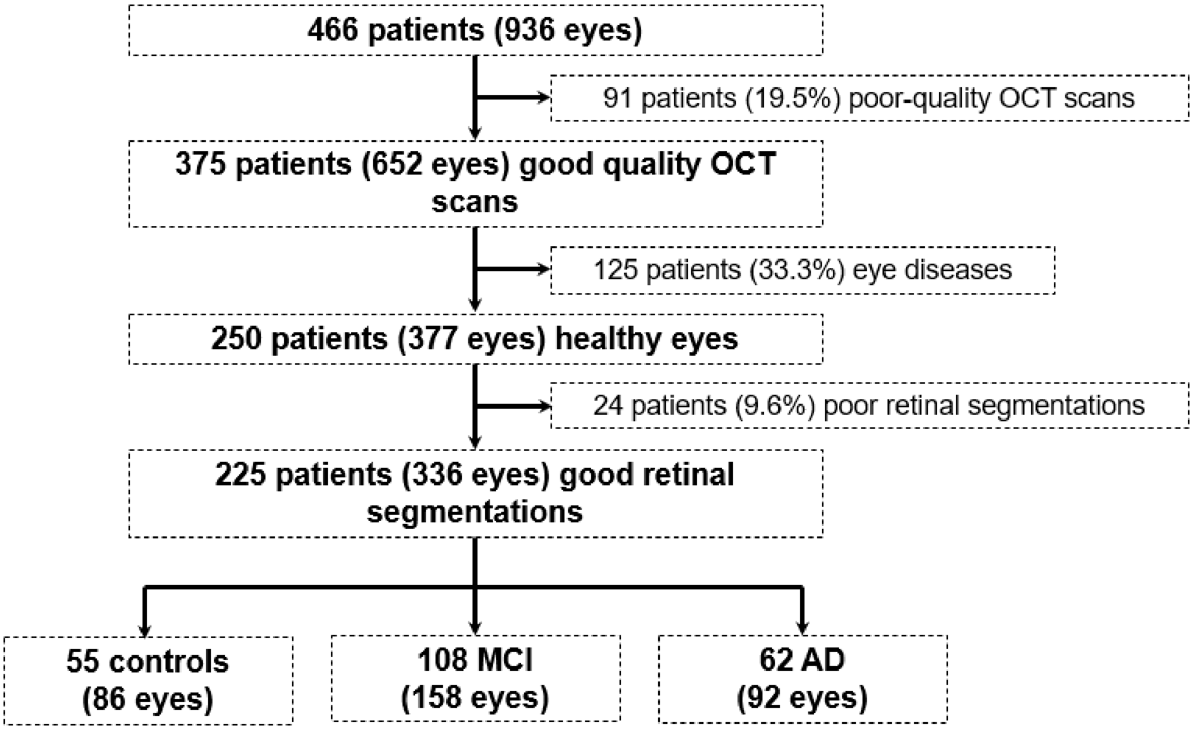


Additional file 2: **Figure S2:** Flow chart indicating the inclusion and exclusion criteria of the study participants, comprising of 55 cognitively normal controls, 108 mild cognitive impairment (MCI) cases, and 62 Alzheimer’s disease (AD) cases.
