# Supplementary material for: A multi-regression framework to improve diagnostic ability of optical coherence tomography retinal biomarkers to discriminate mild cognitive impairment and Alzheimer’s disease
Source: Alzheimers Res Ther. 2022 Mar 10;14:41. doi: 10.1186/s13195-022-00982-0 (PMC8908577; doi:10.1186/s13195-022-00982-0)
Supplement: Supplementary file 3 — Additional file 3. [file 13195_2022_982_MOESM3_ESM.pdf]

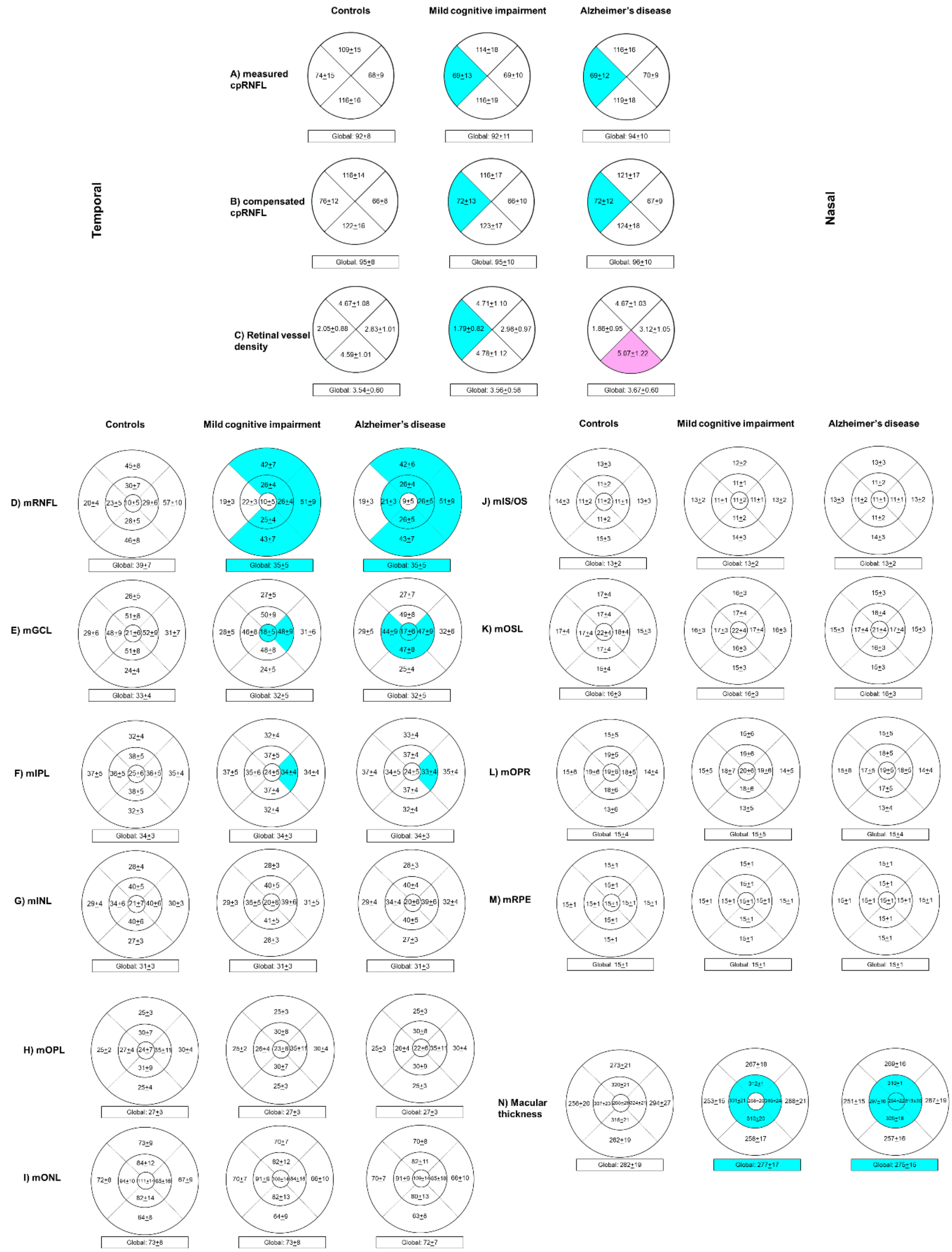

Additional file 3: **Figure S3:** Comparison of thickness measurements of circumpapillary retinal nerve fiber layer (cpRNFL) and retinal vessel density in each quadrant and individual retinal layers in each area of the Early Treatment Diabetic

Retinopathy Study (ETDRS) protocol centered on the fovea in cognitively normal controls, mild cognitive impairment (MCI), and Alzheimer's disease (AD) cases. The data are adjusted for diabetes status, refractive error and represent mean±standard deviation thickness in micrometers. Statistically significant results are shaded in the corresponding sector. Thickening of tissues/vessels is shaded in pink whereas thinning of tissues/vessels are shaded in blue color. In keeping with other studies, changes in retinal layers were only observed in the inner retina. All the graphics are shown as right-eye orientation. Circumpapillary retinal nerve fibre layer (cpRNFL); macular retinal nerve fiber layer (mRNFL); macular ganglion cell layer (mGCL); macular inner plexiform layer (mIPL); macular inner nuclear layer (mINL); macular outer plexiform layer (mOPL); macular outer nuclear layer (mONL); macular inner segments/outer segments layer (mIS/OS); macular outer segment layer (mOSL); macular outer segment PR/RPE complex (mOPR); macular retinal pigment epithelium (mRPE).
